# Dynamic increase in myoglobin level is associated with poor prognosis in critically ill patients: a retrospective cohort study

**DOI:** 10.3389/fmed.2023.1337403

**Published:** 2024-01-08

**Authors:** Yishan Liu, Jinlong Jiang, Hao Yuan, Luhao Wang, Wenliang Song, Fei Pei, Xiang Si, Shumin Miao, Minying Chen, Bin Gu, Xiangdong Guan, Jianfeng Wu

**Affiliations:** ^1^Department of Critical Care Medicine, The First Affiliated Hospital, Sun Yat-sen University, Guangzhou, China; ^2^Guangdong Clinical Research Center for Critical Care Medicine, Guangzhou, China

**Keywords:** myoglobin, critically ill patients, post-surgery, Sepsis, in-hospital motality

## Abstract

**Background:**

Myoglobin is an important biomarker for monitoring critically ill patients. However, the relationship between its dynamic changes and prognosis remains unclear.

**Methods:**

We retrospectively enrolled 11,218 critically ill patients from a general and surgical intensive care unit (ICU) of a tertiary hospital between June 2016 and May 2020. Patients with acute cardiovascular events, cardiac and major vascular surgeries, and rhabdomyolysis were excluded. To investigate the early myoglobin distribution, the critically ill patients were stratified according to the highest myoglobin level within 48 h after ICU admission. Based on this, the critically ill patients with more than three measurements within 1 week after ICU admission were included, and latent class trajectory modeling was used to classify the patients. The characteristics and outcomes were compared among groups. Sensitivity analysis was performed to exclude patients who had died within 72 h after ICU admission. Restricted mean survival time regression model based on pseudo values was used to determine the 28-day relative changes in survival time among latent classes. The primary outcome was evaluated with comparison of in-hospital mortality among each Trajectory group, and the secondary outcome was 28-day mortality.

**Results:**

Of 6,872 critically ill patients, 3,886 (56.5%) had an elevated myoglobin level (≥150 ng/mL) at admission to ICU, and the in-hospital mortality significantly increased when myoglobin level exceeded 1,000 μg/mL. In LCTM, 2,448 patients were unsupervisedly divided into four groups, including the steady group (*n* = 1,606, 65.6%), the gradually decreasing group (*n* = 523, 21.4%), the slowly rising group (*n* = 272, 11.1%), and the rapidly rising group (*n* = 47, 1.9%). The rapidly rising group had the largest proportion of sepsis (59.6%), the highest median Sequential Organ Failure Assessment (SOFA) score (10), and the highest in-hospital mortality (74.5%). Sensitivity analysis confirmed that 98.2% of the patients were classified into the same group as in the original model. Compared with the steady group, the rapidly rising group and the slowly rising group were significantly related to the reduction in 28-day survival time (*β* = −12.08; 95% CI −15.30 to −8.86; *β* = −4.25, 95% CI −5.54 to −2.97, respectively).

**Conclusion:**

Elevated myoglobin level is common in critically ill patients admitted to the ICU. Dynamic monitoring of myoglobin levels offers benefit for the prognosis assessment of critically ill patients.

## Highlights

- Nearly 60% of critically ill patients have increased myoglobin level at admission to intensive care unit.
- Dynamic monitoring of myoglobin, rather than static observation, is beneficial to evaluating the prognosis of critically ill patients.
- Dynamic elevation of myoglobin levels is associated with poor prognosis in critically ill patients.

## Background

Serum concentration of myoglobin is commonly used in the diagnosis and monitoring of acute coronary syndrome (ACS) (1, 2). Since recently, myoglobin has been used in the prognostic assessment of critically ill and postoperative patients (3, 4). A prospective study of critically ill patients without cardiac disease showed that elevated myoglobin level within 24 h of admission was a major risk factor for in-hospital death and 180-day death (5). In addition, myoglobin can be used to predict mortality and conversion to critical illness in severe and critically ill COVID-19 patients, with better efficacy than troponin (6–9). However, there is a lack of data on the distribution of myoglobin levels at the time of ICU admission in critically ill patients.

In critically ill patients, static myoglobin measurements provide limited information, and dynamic changes could offer greater clinical value. Our previous retrospective study showed that myoglobin elevation 14 days after ICU admission was an independent risk factor for death in patients with sepsis-induced chronic critical illness (10). However, that study only observed two time points, and the interval between the two time points was too long to accurately reflect the dynamic changes in myoglobin concentration. Latent class trajectory modeling (LCTM) is a new method to study the trajectory of biomarkers over time, and is now widely used for unsupervised clustering of patients to uncover the potential clinical value of biomakers (11–13). Our previous study based on this model revealed the guiding value of dynamic changes in the lymphocyte count in clinical practice, demonstrating that the model can be used to group critically ill patients (14).

Therefore, the aim of this study was to observe the distribution of myoglobin levels in critically ill patients admitted to the ICU, and to assess the relationship between the dynamic change in myoglobin based on LCTM and the prognosis of critically ill patients.

## Methods

### Ethics approval

This study was performed in strict accordance with the Strengthening the Reporting of Observational Studies in Epidemiology (STROBE) statement (15). Our study was approved by the Independent Ethics Committee for Clinical Research and Animal Trials of The First Affiliated Hospital, Sun Yat-sen University (approval No.: [2022]700). Informed consent was waived as this study did not involve any possible damage to the enrolled patients. The study was registered in China Clinical Trial Registry (Registration No.: ChiCTR2300072040).

### Study design and population

A single-center, retrospective, observational study was conducted in a general and surgical ICU with 56 beds in a tertiary hospital from June 2016 to May 2020. Only the first ICU stay was considered if a patient had multiple ICU admissions. Patients with absence of case data, age less than 18 years, acute cardiovascular events during hospitalization (successful resuscitation after respiratory and cardiac arrest, acute coronary syndrome, aortic dissection, pulmonary embolism), cardiac and great vessel surgery, and rhabdomyolysis were excluded. Only adult patients with myoglobin measurement within 48 h after entering the ICU were included to describe the distribution of myoglobin levels, and patients with at least three myoglobin measurements within 7 days after ICU admission were included for trajectory.

### Data collection and evaluation

Demographic characteristics, clinical parameters, and laboratory examination results were extracted from the electronic databases of hospital information system and ICU system. The maximum myoglobin measurement before entering the ICU was taken as baseline value, and only the maximum value was retained for multiple measurements within the same day (13). Other laboratory indicators, including creatine kinase MB (CK-Mb), pro-B-type natriuretic peptide (pro-BNP), and cardiac troponin I (cTnI), were also collected. In addition, the maximum values of lactate, total bilirubin, and creatinine, the lowest urine volume, and mean arterial pressure (MAP) were recorded 72 h after entering the ICU to evaluate the tissue perfusion and organ functional status of the patients.

In-hospital death was determined based on the information in the electronic database (extracted from the home page of the medical record). Sepsis and Sequential Organ Failure Assessment (SOFA) scores were defined according to sepsis 3.0 (16). During assessment, the Glasgow Coma Scale (GCS) score was found to be subjective and heterogeneous; hence, it was removed from the SOFA score. Acute kidney injury (AKI) was diagnosed in line with the Kidney Disease Improving Global Outcomes (KDIGO) criteria (17). ACS (ST-segment elevation myocardial infarction, non-ST-segment elevation myocardial infarction, and unstable angina pectoris) was diagnosed by cardiovascular physicians based on electrocardiographic findings, troponin level, and previous cardiac conditions. Other major diagnoses were made based on the codes of the International Classification of Diseases (ICD), Ninth Edition. Other ICU treatment records were derived from the electronic database (extracted from the ICU system).

Two practitioners were responsible for assessing the integrity and quality of data of the included patients. The data integrity was evaluated mainly for the in-hospital survival. The quality assessment included the diagnostic accuracy of sepsis and AKI to control for the disease misclassification. Simultaneously, sampling quality control was carried out by two senior physicians.

### Statistical method

Based on myoglobin levels (ng/mL) within 48 h after ICU admission, the patients were divided into four groups, namely normal (<150), mild increase (150–500), middle increase (500–1,000), and severe increase (≥1,000), consistent with the cutoff values previously utilized by other authors (18, 19). Differences between the groups were analyzed. The primary outcome was evaluated with comparison of in-hospital mortality among each Trajectory group, and the secondary outcome was 28-day mortality. Percentages were used to describe discrete variables, and chi-squared or Fisher’s exact test was applied to test hypothesis. Medians and interquartile ranges (IQRs) were used to describe continuous variables, and Kruskal–Wallis rank-sum test was applied to test hypothesis among the four groups. In LCTM analysis, the population was classified based on daily myoglobin examinations from before ICU admission to 7 days after ICU admission. Since there were very few maxima of myoglobin concentration, the examination values were transformed logarithmically. The optimal number of latent classes was determined by relative entropy, Bayesian Information Criterion (BIC), and the proportion of each latent class. The percentages of subjects classified with a posterior probability above 0.7/0.8/0.9 and the table of posterior classification were also demonstrated. Based on BIC and relative entropy, we finally selected the number of 4. The 28-day Kaplan–Meier (KM) curve was created, and log-rank test was applied to test the hypothesis. To verify the robustness of the above model in different populations, subpopulations with or without sepsis, and with or without surgery were filtered to re-fit the LCTM algorithm, and the corresponding KM curves were created. In sensitivity analysis, patients with survival time less than or equal to 72 h were excluded. The consistency between the sensitivity analysis and the original analysis was calculated. To intuitively demonstrate the effect of covariates on the patients’ survival time, restricted mean survival time (RMST) regression based on pseudo values was used to assess the differences in 28-day RMST (20). Age over 65 years, sex, SOFA score, sepsis, chronic kidney disease (CKD), hepatic disease, post-surgery, and minimum MAP within 72 h were incorporated into the regression as confounders. Forest plot was also created. Random-forest missing-value imputation method was applied to impute missing values (21). In this study, missing value imputation was only applied in regression analysis. Detailed description of LCTM and RMST regression is shown in Supplementary Statistical Methods. All statistical analyses were performed with R 4.2.1, and two-sided *p* < 0.05 was considered statistically significant. Bonferroni’s correction was applied for multiple comparisons.

## Results

### Distribution of myoglobin levels in critically ill patients at admission to the ICU

**Figure 1:**
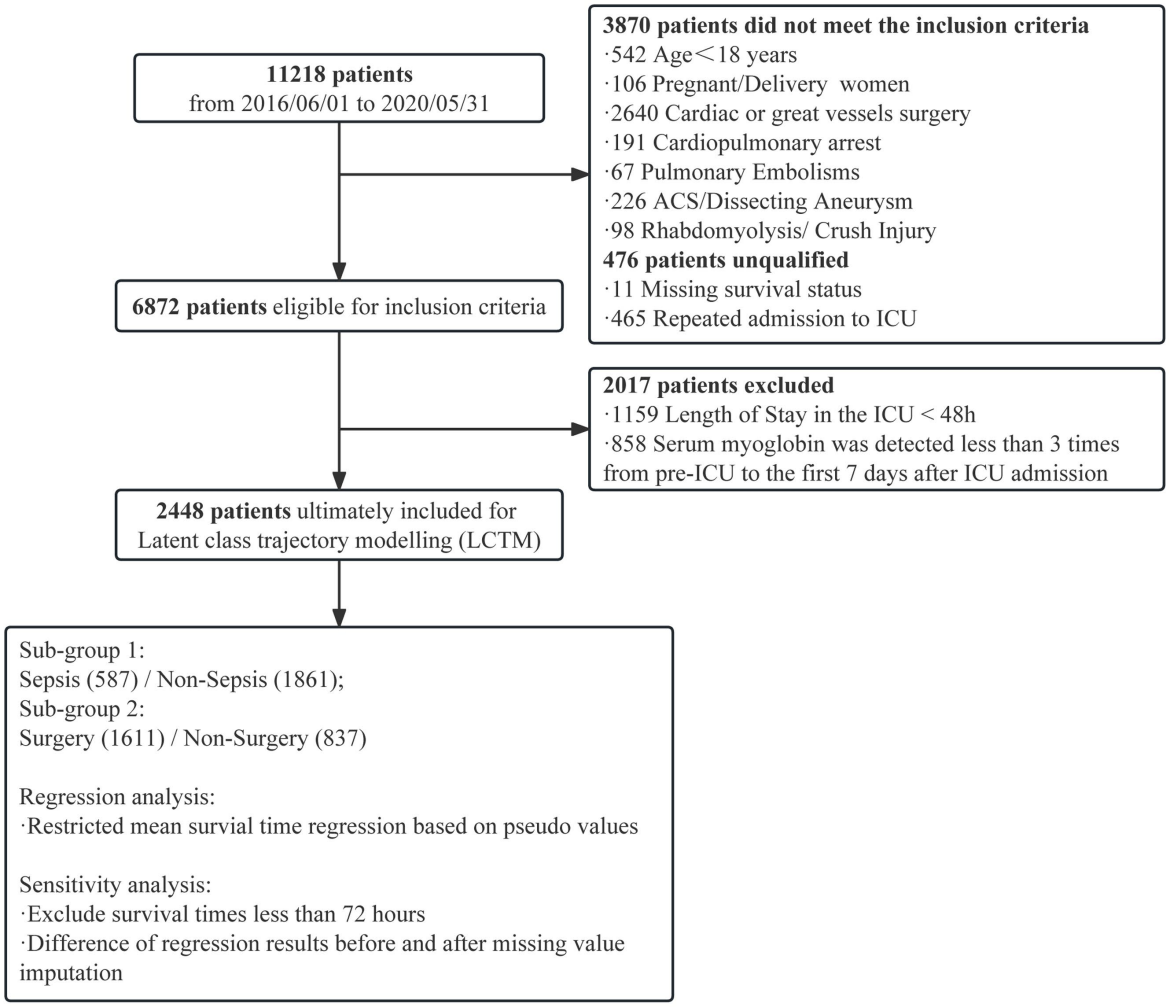
Flowchart of the study. ACS: Acute coronary syndrome. ICU: Intensive care unit.

**Figure 2:**
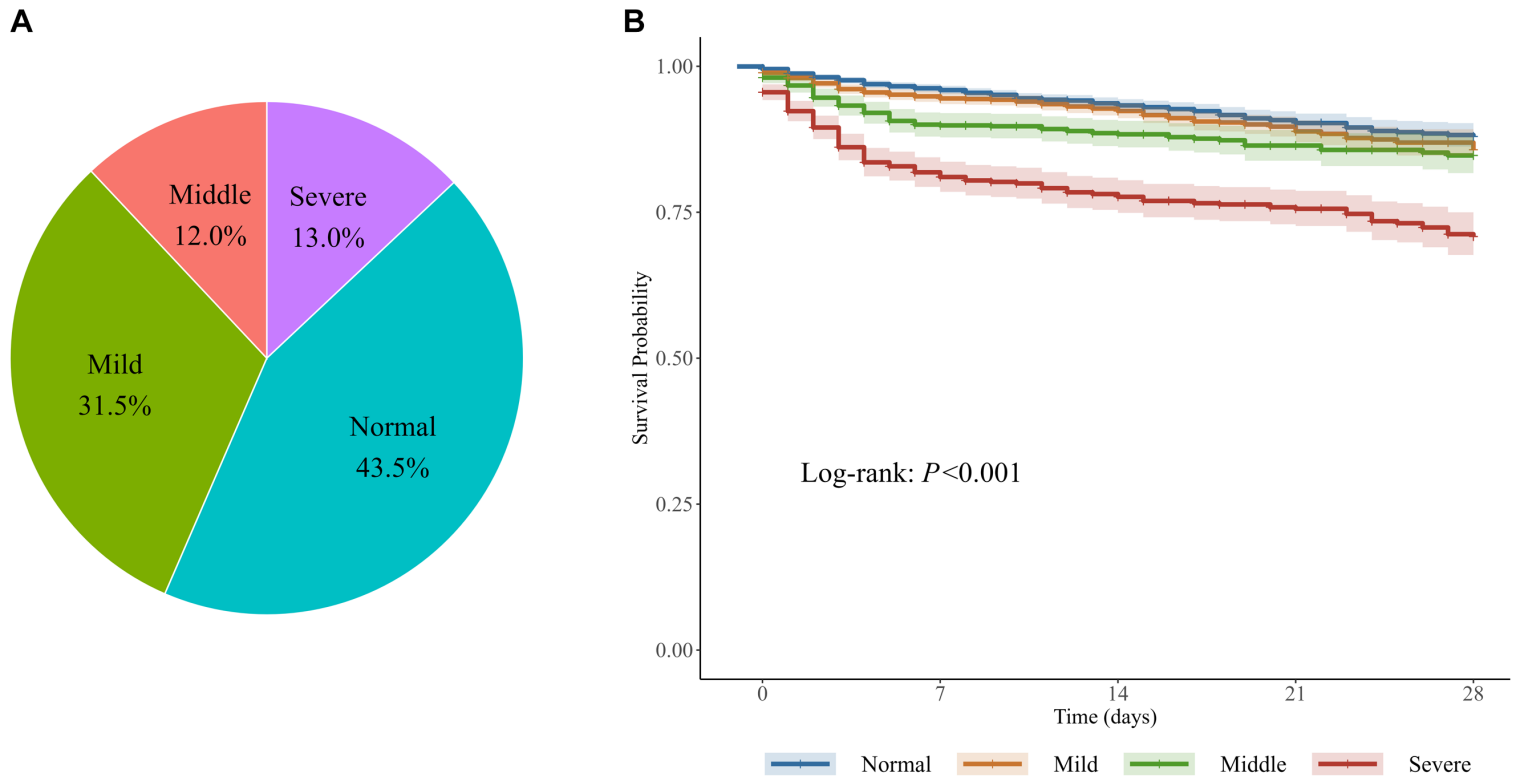
Serum myoglobin levels after admitted to the ICU and their relationship with mortality. **(A)** The constituent ratio of 4 groups classified by myoglobin levels within 48 hours after admitted to the ICU. **(B)** The corresponding Kaplan-Meier curve in patients with elevated myoglobin. 6,872 patients were divided into four groups based on myoglobin (ng/ml) within 48 hours after ICU admission: Normal (< 150), Mild increasing (150~499), Middle increasing (500~999), Severe increasing (≥ 1000).

A total of 6,872 patients were included to describe the distribution of myoglobin concentrations (Figure 1). The median age of the patients was 54 years, 58.6% were male, the overall in-hospital mortality rate was 10.1%, and the median SOFA score was 7 points. Of the enrolled patients, 56.5% (*n* = 3,886) had elevated myoglobin concentration when entering the ICU, including 31.5% with mild increase, 12.0% with middle increase, and 13.0% with severe increase (Figure 2A). There was no difference in the proportion of patients with past medical history of chronic cardiovascular diseases among the groups. As shown in Table 1, the severe increasing group showed significantly higher proportion of sepsis (19.7% vs. 8.7%, *p* < 0.001), in-hospital mortality (24.2% vs. 6.6%, *p* < 0.001), and use of vasoactive drugs (31.9% vs. 64.3%, *p* < 0.001) than the normal group. Only the patients in the severe increasing group had a higher 28-day mortality than those in the normal group (Figure 2B).

**Table 1:** Characteristics of critically ill patients with different myoglobin groups.

Characteristic	Overall, *N* = 6,872^1^	Normal, *N* = 2,986^1^	Mild, *N* = 2,163^1^	Middle, *N* = 823^1^	Severe, *N* = 900^1^	*P* value^2^
Patients’ characteristics						
Age	54.0 (43.0, 65.0)	54.0 (41.0, 65.0)	55.0 (44.0, 66.0)	55.0 (45.0, 64.0)	52.0 (41.0, 63.0)	<0.001
Sex (Male)	4,025 (58.6%)	1,444 (48.4%)	1,287 (59.5%)	595 (72.3%)	699 (77.7%)	<0.001
SOFA score^3^	7.0 (6.0, 8.0)	6.0 (5.0, 7.0)	6.0 (6.0, 8.0)	7.0 (6.0, 9.0)	8.0 (6.0, 10.0)	<0.001
Vasopressors in ICU	2,839 (41.3%)	953 (31.9%)	820 (37.9%)	487 (59.2%)	579 (64.3%)	<0.001
Vasopressors use duration (hours)	30.7 (24.0, 114.4)	32.1 (24.0, 125.3)	24.9 (24.0, 104.5)	27.4 (24.0, 87.9)	37.5 (23.3, 121.8)	<0.001
AKI^4^	894 (13.4%)	251 (8.7%)	256 (12.1%)	142 (17.8%)	245 (28.0%)	<0.001
CRRT within first 24 h	510 (7.4%)	110 (3.7%)	156 (7.2%)	70 (8.5%)	174 (19.3%)	<0.001
Admission types						
Underlying disease						<0.001
Yes	4,007 (58.3%)	1,589 (53.2%)	1,255 (58.0%)	584 (71.0%)	579 (64.3%)	
No	2,865 (41.7%)	1,397 (46.8%)	908 (42.0%)	239 (29.0%)	321 (35.7%)	
Sepsis						<0.001
Yes	751 (10.9%)	259 (8.7%)	228 (10.5%)	87 (10.6%)	177 (19.7%)	
No	6,121 (89.1%)	2,727 (91.3%)	1,935 (89.5%)	736 (89.4%)	723 (80.3%)	
Post-surgery						<0.001
Yes	5,682 (82.7%)	2,406 (80.6%)	1,858 (85.9%)	713 (86.6%)	705 (78.3%)	
No	1,190 (17.3%)	580 (19.4%)	305 (14.1%)	110 (13.4%)	195 (21.7%)	
Past medical history						
Hypertension	1,462 (21.3%)	653 (21.9%)	468 (21.6%)	160 (19.4%)	181 (20.1%)	0.365
Coronary artery disease	234 (3.4%)	88 (2.9%)	92 (4.3%)	29 (3.5%)	25 (2.8%)	0.052
Chronic heart failure	139 (2.0%)	69 (2.3%)	41 (1.9%)	16 (1.9%)	13 (1.4%)	0.396
Valvular disease	198 (2.9%)	105 (3.5%)	49 (2.3%)	22 (2.7%)	22 (2.4%)	0.046
Chronic bronchitis/Chronic obstructive pulmonary disease	143 (2.1%)	76 (2.5%)	43 (2.0%)	11 (1.3%)	13 (1.4%)	0.064
Viral hepatitis	141 (2.1%)	47 (1.6%)	22 (1.0%)	36 (4.4%)	36 (4.0%)	<0.001
Cirrhosis	268 (3.9%)	38 (1.3%)	55 (2.5%)	90 (10.9%)	85 (9.4%)	<0.001
Hepatic failure	179 (2.6%)	39 (1.3%)	49 (2.3%)	32 (3.9%)	59 (6.6%)	<0.001
CKD	229 (3.3%)	47 (1.6%)	98 (4.5%)	37 (4.5%)	47 (5.2%)	<0.001
Malignancy	2,465 (35.9%)	922 (30.9%)	818 (37.8%)	402 (48.8%)	323 (35.9%)	<0.001
Respiratory characteristics						
MV in ICU	5,659 (82.3%)	2,208 (73.9%)	1,880 (86.9%)	738 (89.7%)	833 (92.6%)	<0.001
Duration of MV (hours)	7.0 (2.0, 25.0)	4.0 (2.0, 19.0)	5.0 (2.0, 19.0)	12.0 (4.0, 36.0)	15.0 (4.0, 73.0)	<0.001
Ventilator-free days at Day 28	27.9 (27.2, 28.0)	27.9 (27.5, 28.0)	27.9 (27.2, 28.0)	27.5 (26.3, 27.9)	27.4 (12.0, 27.9)	<0.001
Clinical outcomes						
ICU duration (hours)	40.8 (21.6, 108.2)	37.9 (21.8, 111.1)	31.7 (21.1, 90.9)	45.5 (22.9, 108.0)	56.7 (24.1, 138.2)	<0.001
Length of hospital stay(days)	20.0 (14.0, 30.0)	19.0 (13.0, 27.0)	21.0 (14.0, 29.0)	23.0 (16.0, 36.0)	23.5 (13.0, 37.0)	<0.001
In-hospital death	696 (10.1%)	196 (6.6%)	180 (8.3%)	102 (12.4%)	218 (24.2%)	<0.001

CKD, Chronic Kidney Diseases; MV, Mechanical Ventilation; SOFA, Sepsis-related Organ Failure Assessment; GCS, Glasgow coma score. ^1^Median (IQR); n (%); ^2^Kruskal-Wallis rank sum test; Pearson’s Chi-squared test; ^3^SOFA score: without GCS score; ^4^AKI, Acute Kidney Injury, based on KIDGO criteria, only Grade 2 or Grade 3 were included.

### Trajectories of myoglobin concentration in critically ill patients

A total of 2,448 patients were enrolled in myoglobin trajectory modeling (Figure 1). The median age of the patients was 59.0 years, 66.7% were male, the overall in-hospital mortality rate was 17.8%, and the median SOFA score was 8.0.

**Figure 3:**
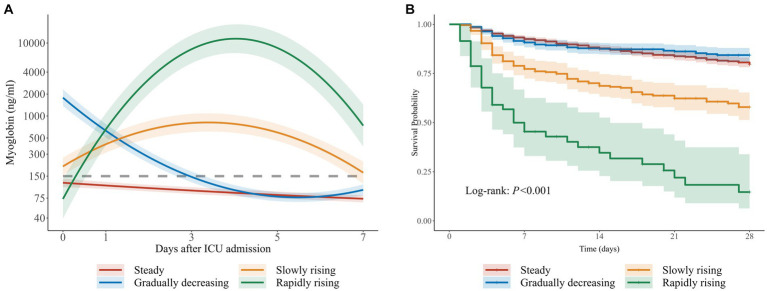
Trajectories of myoglobin in critically ill patients. **(A)** Four trajectories of myoglobin in 2,448 critically ill patients: Hereinafter referred to as steady group, gradually decreasing group, slowly rising group and rapidly rising group. The gray dotted line represents 150ng/ml. **(B)** The corresponding Kaplan-Meier curve of four groups.

Considering the BIC and relative entropy of the trajectory model, four groups were finally determined (Supplementary Tables 1–3). The four trajectory groups were as follows: Group 1 (65.6%, *n* = 1,606), slight fluctuation of myoglobin level within the normal range (hereinafter referred to as the steady group); Group 2 (21.4%, *n* = 523), initially high myoglobin level rapidly decreased to normal (the gradually decreasing group); Group 3 (11.1%, *n* = 272), myoglobin level slightly increased and then decreased slowly (the slowly rising group); and Group 4 (1.9%, *n* = 47), myoglobin level rapidly increased and then decreased gradually (the rapidly rising group). All dynamic trajectories are shown in Figure 3A. In the sensitivity analysis, the individuals whose survival time was less than or equal to 72 h were excluded, and a new LCTM was fitted (Supplementary Figure 1). The new model classified 98.2% of the individuals in the same way as the original model. In the new model, the proportion of patients in the same group was 92.7% in the rapidly rising group and 95.9% in the slowly rising group.

The characteristics and outcomes of the patients based on LCTM are shown in Table 2. In terms of demographic characteristics, the median age in the rapidly rising group and the steady group was higher than that in the gradually decreasing group and the slowly rising group. There was a significant difference in gender distribution among the four groups (*p* < 0.001), with lower proportion of male patients in the steady group than in the other three groups. The proportions of chronic cardiovascular diseases, diabetes, and CKD were lower in the rapidly rising group than in the steady group, but the rapidly rising group had the highest SOFA score, lactic acid, and bilirubin, the lowest MAP, and the highest incidence of AKI and sepsis. In terms of outcomes, there was a significant difference in 28-day mortality among the groups (*p* < 0.001). In the gradually decreasing group, myoglobin level was the highest at admission to the ICU, and the in-hospital mortality was the lowest among all groups. The in-hospital mortality in the slowly rising group and rapidly rising group was higher than that in the steady group. There was no significant difference in the in-hospital mortality between the steady group and the gradually decreasing group.

**Table 2:** Characteristics of critically ill patients among trajectory groups.

Characteristic	Overall, *N* = 2,448^1^	Steady, *N* = 1,606^1^	Gradually decreasing, *N* = 523^1^	Slowly rising, *N* = 272^1^	Rapidly rising, *N* = 47^1^	*P* value^2^
Patients’ characteristics						
Age	59.0 (47.0, 68.0)	60.0 (48.0, 70.0)	54.0 (44.0, 65.0)	58.0 (46.0, 68.0)	61.0 (46.5, 70.5)	<0.001
Sex (Male)	1,632 (66.7%)	991 (61.7%)	393 (75.1%)	213 (78.3%)	35 (74.5%)	<0.001
SOFA score^3^	8.0 (6.0, 9.0)	7.0 (6.0, 9.0)	8.0 (6.0, 9.0)	9.0 (8.0, 10.0)	10.0 (8.5, 11.0)	<0.001
Vasopressors in ICU	1,808 (73.9%)	1,109 (69.1%)	412 (78.8%)	240 (88.2%)	47 (100.0%)	<0.001
Min MAP in the first 3 days	58.7 (50.0, 66.0)	59.0 (52.0, 66.0)	59.0 (50.3, 66.0)	55.0 (45.0, 64.0)	46.7 (31.0, 54.8)	<0.001
Max Lac in the first 3 days (mmol/L)	2.8 (1.8, 4.9)	2.5 (1.7, 4.1)	3.0 (2.0, 5.4)	4.4 (2.5, 9.2)	14.2 (8.8, 15.0)	<0.001
AKI^4^	676 (28.9%)	396 (25.9%)	95 (18.9%)	147 (56.1%)	38 (84.4%)	<0.001
Mean urine per hour in the first 3 days (ml)	104.1 (76.8, 134.0)	104.4 (79.8, 135.1)	115.2 (87.7, 142.7)	83.7 (22.8, 118.1)	24.9 (10.5, 64.2)	<0.001
Max Cr in the first 3 days (mmol/L)	93.0 (67.0, 164.8)	86.0 (62.0, 140.0)	93.0 (69.0, 157.0)	161.0 (99.5, 280.5)	194.0 (151.0, 262.0)	<0.001
CRRT within first 24 h	398 (16.3%)	211 (13.1%)	54 (10.3%)	104 (38.2%)	29 (61.7%)	<0.001
Max TBil in the first 3 days (mmol/L)	23.1 (14.4, 54.6)	20.2 (13.2, 40.7)	29.4 (16.0, 75.1)	40.7 (19.7, 123.2)	106.5 (32.5, 272.3)	<0.001
Mb of Day 1 (ng/ml)	207.8 (74.6, 655.2)	115.4 (51.8, 245.0)	828.2 (428.2, 1,566.0)	738.4 (263.2, 1,842.0)	762.0 (238.3, 3,345.0)	<0.001
Mb of Day 2 (ng/ml)	170.2 (64.7, 485.1)	97.0 (46.5, 237.1)	347.6 (155.1, 786.0)	941.5 (410.6, 2,508.0)	2,905.0 (1,169.0, 9,829.0)	<0.001
Mb of Day 3 (ng/ml)	120.2 (49.4, 351.4)	82.2 (38.4, 188.1)	151.5 (64.8, 335.5)	924.8 (462.0, 2,227.5)	12,197.5 (5,313.0, 27,516.2)	<0.001
Admission types						
Underlying disease						<0.001
Yes	1,687 (68.9%)	1,091 (67.9%)	343 (65.6%)	215 (79.0%)	38 (80.9%)	
No	761 (31.1%)	515 (32.1%)	180 (34.4%)	57 (21.0%)	9 (19.1%)	
Sepsis						<0.001
Yes	587 (24.0%)	366 (22.8%)	104 (19.9%)	89 (32.7%)	28 (59.6%)	
No	1,861 (76.0%)	1,240 (77.2%)	419 (80.1%)	183 (67.3%)	19 (40.4%)	
Post-surgery						<0.001
Yes	1,611 (65.8%)	997 (62.1%)	403 (77.1%)	181 (66.5%)	30 (63.8%)	
No	837 (34.2%)	609 (37.9%)	120 (22.9%)	91 (33.5%)	17 (36.2%)	
Past medical history						
Hypertension	696 (28.4%)	502 (31.3%)	105 (20.1%)	82 (30.1%)	7 (14.9%)	<0.001
Coronary artery disease	139 (5.7%)	111 (6.9%)	15 (2.9%)	12 (4.4%)	1 (2.1%)	0.002
Chronic heart failure	91 (3.7%)	72 (4.5%)	9 (1.7%)	10 (3.7%)	0 (0.0%)	0.013
Valvular disease	106 (4.3%)	80 (5.0%)	12 (2.3%)	14 (5.1%)	0 (0.0%)	0.019
Chronic bronchitis/Chronic obstructive pulmonary disease	86 (3.5%)	70 (4.4%)	8 (1.5%)	8 (2.9%)	0 (0.0%)	0.008
Viral hepatitis	67 (2.7%)	44 (2.7%)	10 (1.9%)	12 (4.4%)	1 (2.1%)	0.225
Cirrhosis	139 (5.7%)	54 (3.4%)	59 (11.3%)	25 (9.2%)	1 (2.1%)	<0.001
Hepatic failure	113 (4.6%)	56 (3.5%)	21 (4.0%)	24 (8.8%)	12 (25.5%)	<0.001
CKD	152 (6.2%)	105 (6.5%)	21 (4.0%)	26 (9.6%)	0 (0.0%)	0.004
Malignancy	903 (36.9%)	550 (34.2%)	218 (41.7%)	114 (41.9%)	21 (44.7%)	0.003
Respiratory characteristics						
MV in ICU	1,860 (76.0%)	1,120 (69.7%)	453 (86.6%)	244 (89.7%)	43 (91.5%)	<0.001
Duration of MV (hours)	55.0 (13.0, 139.0)	53.5 (12.0, 152.2)	36.0 (12.0, 104.0)	88.0 (32.5, 151.5)	66.0 (37.5, 173.0)	<0.001
Ventilator-free days at Day 28	27.0 (20.1, 28.0)	27.3 (21.8, 28.0)	27.1 (22.6, 27.8)	23.1 (1.5, 27.1)	1.7 (0.7, 21.3)	<0.001
Clinical outcomes						
ICU duration (hours)	139.0 (86.5, 261.9)	143.8 (88.1, 270.6)	118.7 (83.7, 220.3)	142.4 (86.9, 277.6)	119.6 (77.2, 196.9)	0.015
Length of hospital stay(days)	23.0 (13.0, 37.0)	22.0 (13.0, 35.0)	25.0 (16.0, 42.0)	22.0 (12.0, 37.0)	14.0 (7.0, 24.0)	<0.001
In-hospital death	435 (17.8%)	233 (14.5%)	71 (13.6%)	96 (35.3%)	35 (74.5%)	<0.001

CKD: Chronic Kidney Diseases, MV: Mechanical Ventilation, SOFA: Sepsis-related Organ Failure Assessment, GCS: Glasgow coma score. ^1^Median (IQR); n (%); ^2^Kruskal-Wallis rank sum test; Pearson’s Chi-squared test; Fisher’s exact test; ^3^SOFA score: without GCS score; ^4^AKI: Acute Kidney Injury, based on KIDGO criteria, only Grade 2 or Grade 3 were included.

To evaluate the stability of the model in different subgroups, we further studied model establishment in sepsis/non-sepsis (*n* = 587/1,861) and surgery/non-surgery (*n* = 1,611/837) patients based on previous investigation (22–24). The trajectory plots and the corresponding KM curves are shown in Supplementary Figures 2, 3. In the above four subgroups, the myoglobin trajectories and the trend of the KM curves were both similar to the overall pattern.

### Relationship among the trajectory groups and the expected 28-day survival time

**Figure 4:**
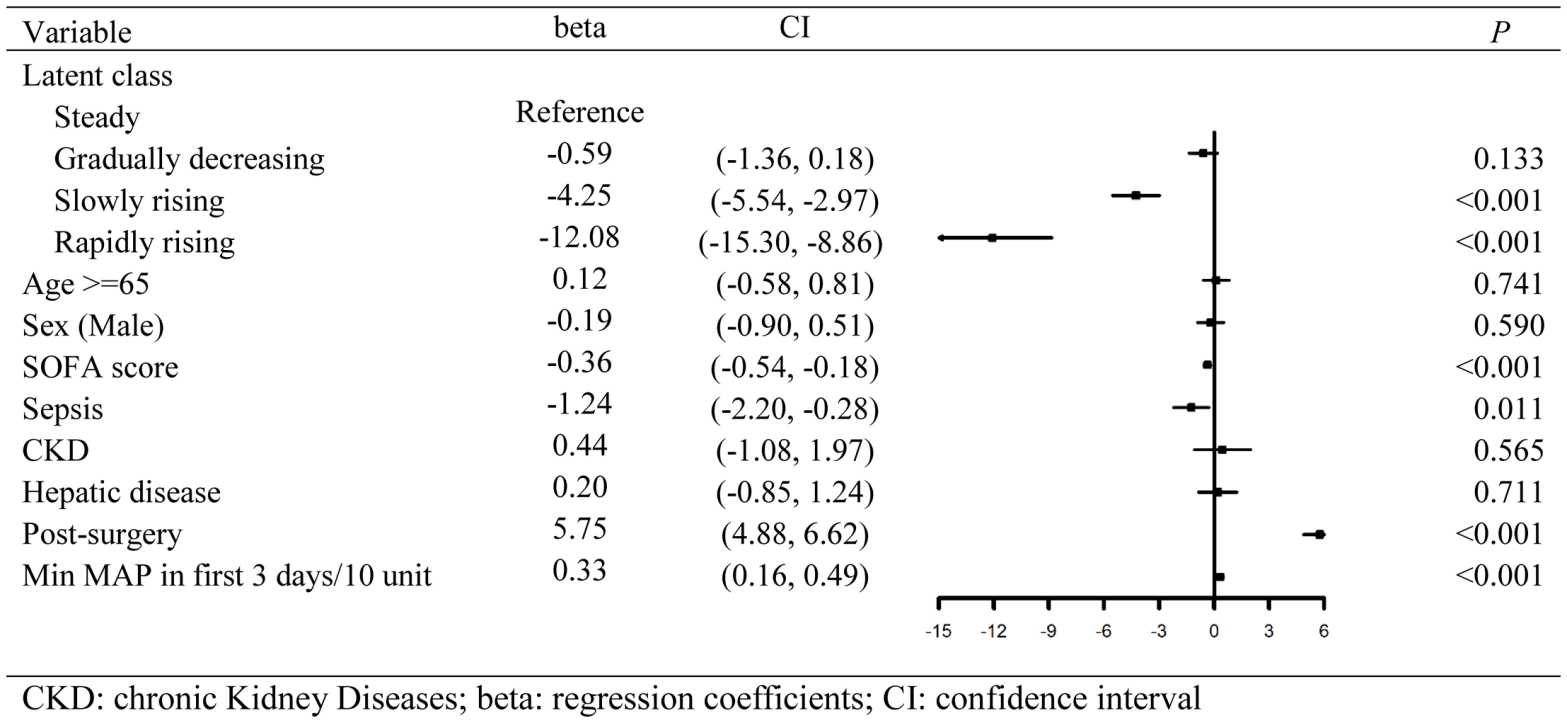
Forest plot of multivariate regression results calculated by restricted mean survival time.

With the steady group as the reference, RMST regression based on pseudo values was employed to evaluate the relative changes in the expected 28-day survival time between different trajectory groups. Multivariate regression results are shown in Figure 4. After adjustment for gender, age, and other confounders, there was no statistically significant difference in the expected 28-day RMST between the gradually decreasing group and the steady group. However, compared with the expected 28-day RMST of the steady group, that of the slowly rising group and the rapidly rising group decreased by 4.25 days and 12.08 days, respectively (*p* < 0.001).

Of 2,448 patients, 12.7% had missing values, which were deleted in the RMST regression. The regression results after filling the missing values by a random-forest algorithm were similar to those without imputation (Supplementary Figure 4).

## Discussion

In this retrospective observational study, more than half of the critically ill patients had an increased myoglobin level (≥150 μg/mL) when admitted to the ICU, and the in-hospital mortality significantly increased when myoglobin exceeded 1,000 μg/mL. Our study further showed that the 28-day mortality in the gradually decreasing group was similar to that in the stable group, but the survival time of the patients with dynamic myoglobin increase (the slowly rising group and the rapidly rising group) significantly decreased relative to that of the stable group. In general, elevated myoglobin level is a common phenomenon among critically ill patients, and dynamic monitoring of myoglobin levels can help to evaluate the prognosis of critically ill patients.

Previous studies have shown that myoglobin elevation is related to the severity and prognosis of critically ill patients; however, the incidence of myoglobin elevation remains unclear (3, 4, 25). In this four-year retrospective study, 56.5% of the critically ill patients showed increased myoglobin levels (≥150 μg/mL). Our results were similar to those of a previous study with 179 critically ill patients, which reported that 40.8% of the critically ill patients without ACS had elevated myoglobin level and 18 (10%) patients presented severe myoglobin elevation (≥1,000 μg/mL) on admission to the ICU (5). The previous study included critically ill patients without surgery, while in our study, two-thirds were postsurgical patients. Therefore, our results can be widely applied to critically ill patients.

Interestingly, the lowest in-hospital mortality was observed in the critically ill patients from the gradually decreasing group with the highest myoglobin level at admission to the ICU. This seems to be inconsistent with the findings of increased mortality with severe elevated myoglobin on admission to the ICU. The same trend was noticed in the RMST regression, and the expected 28-day RMST of the gradually decreasing group was statistically equivalent to that of the steady group. However, compared with the expected 28-day RMST of the steady group, that of the slowly rising group and the rapidly rising group decreased by 4.25 days and 12.08 days, respectively. Therefore, according to our study, myoglobin measurement at a single time point provides weak evidence, and the dynamic change of myoglobin level is more significant for the prognosis. In our previous retrospective study, 131 patients with sepsis were divided into the myoglobin rise group and the myoglobin fall group according to the myoglobin levels on the first and 14th days (10). The 28-day cumulative survival rate of the rise group was significantly lower than that of the fall group. Therefore, static myoglobin evaluation does not effectively reflect the prognosis of critically ill patients, and dynamic monitoring of myoglobin levels is recommended.

The reason for the dynamic increase in myoglobin in critically ill patients is not fully understood, and it should be examined why myoglobin leads to poor prognosis of critically ill patients. In the RMST regression, after adjustment for age, sepsis, SOFA score, and other confounders, the increase in myoglobin level was still significantly related to the decrease in the survival time. Previous studies have documented that hypoperfusion and infection can lead to myocardial injury, a nonischemic myocardial injury without characteristic electrocardiogram changes, in critically ill patients (23, 26, 27). Although we attempted to exclude patients with potential myocardial injury, such as cardiac arrest, ACS, and pulmonary embolism, the lower median MAP and urine volume and higher median lactate in the patients of two rising groups suggest poor tissue and organ perfusion in these patients. In addition, according to the retrospective analysis of other myocardial injury markers such as cTnI, CK-Mb, and pro-BNP (Supplementary Table 4) of the four groups of patients, a trend of continuous increase in these markers was observed in the rapidly rising and slowly rising groups. Animal research has shown that skeletal muscle is less sensitive to hypoperfusion than myocardium, and there is no significant increase in myoglobin release from skeletal muscle under hypoperfusion (28). Hence, myocardial injury is likely one of the main causes of myoglobin elevation in critically ill patients. However, these patients are difficult to be diagnosed as ACS and septic cardiomyopathy during hospitalization in the ICU (26, 29, 30). Our study suggests that dynamic monitoring of myoglobin levels may be helpful for identification of critically ill patients with latent myocardial injury.

Renal dysfunction may be another important factor causing the dynamic increase in myoglobin level. Our results showed that the incidence of AKI was very high in the rapidly rising group and the slowly rising group, while it was only 18.9% in the gradually decreasing group, which was the lowest among the four groups. The patients in the gradually decreasing group had excellent baseline renal function, revealing a very low proportion of CKD. Under normal circumstances, myoglobin is excreted through kidneys, and it returns to the normal range within 20–30 h in patients with acute myocardial infarction (31). Renal dysfunction injury leads to the excretion disorder of myoglobin, resulting in the increased level of myoglobin. The increased myoglobin concentration can also aggravate renal dysfunction (32, 33). Hence, acute or chronic renal dysfunction and myoglobin increase are causally related, and special attention should be paid to the influence of renal function on the dynamic changes of myoglobin in clinical monitoring. Therefore, dynamic change of myoglobin level can help to predict the prognosis of critically ill patients (Supplementary Tables 5, 6).

Our study has several limitations. First, in LCTM, the minimum percentage of each subgroup is recommended to be 7% (34). If only three groups of trajectories had been fitted, the rapidly rising group and the slowly rising group would have been merged, which might restrict further identification of the difference in survival rate. Through further fitting based on subgroups with/without sepsis and surgery, similar population distribution and survival were observed, supporting a robust trajectory modeling in our study. Second, there was a large proportion of patients after operation in the cohort, with the lack of information about the duration and position of operation, which has been identified to be the potential cause of myoglobin increase in patients (35). Third, designed as a retrospective observational survey, our study failed to fully collect data related to confounding factors. Therefore, the causal relationship between the latent class trajectory of myoglobin and survival time reduction should be considered with caution in a strict sense. Fourth, subject to the limitation of retrospective study, our study cannot clarify the causal relationship between the elevated myoglobin levels and the severity of critically ill patients. Further prospective studies are in great need to investigate the detailed relationship between the elevated myoglobin levels and the prognosis of critically ill patients.

## Conclusion

Elevated myoglobin level is common in critically ill patients admitted to the ICU. Dynamic monitoring of myoglobin offers benefit to the prognosis assessment of critically ill patients.

## Data availability statement

The original contributions presented in the study are included in the article/Supplementary material, further inquiries can be directed to the corresponding author.

## Ethics statement

The studies involving humans were approved by Independent Ethics Committee for Clinical Research and Animal Trials of The First Affiliated Hospital, Sun Yat-sen University (approval No.: [2022]700). The studies were conducted in accordance with the local legislation and institutional requirements. The participants provided their written informed consent to participate in this study.

## Author contributions

YL: Writing – original draft. JJ: Writing – original draft. HY: Writing – original draft. LW: Data curation, Writing – original draft. WS: Writing – original draft. FP: Writing – original draft, Writing – review & editing. XS: Writing – review & editing. SM: Writing – review & editing. MC: Writing – review & editing. BG: Writing – review & editing. XG: Writing – review & editing. JW: Writing – review & editing.

## Supplementary material

SUPPLEMENTARY FIGURE 1 Trajectories of myoglobin for sensitivity analysis. Individuals whose survival time was over than 72 hours were preserved in the sensitivity analysis. The gray dotted line represents 150 ng/ml.

SUPPLEMENTARY FIGURE 2 Trajectory plots of subgroups: **(A)** Sepsis, **(B)** Non-Sepsis, **(C)** Surgery, **(D)** Non-Surgery.

SUPPLEMENTARY FIGURE 3 Kaplan-Meier curves of sub-groups: **(A)** Sepsis, **(B)** Non-Sepsis, **(C)** Surgery, **(D)** Non-Surgery.

SUPPLEMENTARY FIGURE 4 Forest plot of multivariate regression results calculated by restricted mean survival time based on pseudo values after missing value imputation.
