# Supplementary material for: Dynamic increase in myoglobin level is associated with poor prognosis in critically ill patients: a retrospective cohort study
Source: Front Med (Lausanne). 2024 Jan 8;10:1337403. doi: 10.3389/fmed.2023.1337403 (PMC10804859; doi:10.3389/fmed.2023.1337403)
Supplement: Supplementary file 1 [file Table_1.docx]

**Supplementary Table 1** Fit statistics for different number of trajectory groups

| Num. of latent class | Relative entropy | BIC | Percentage of each latent class (%) | | | | | | |
| --- | --- | --- | --- | --- | --- | --- | --- | --- | --- |
|  |  |  | 1 | 2 | 3 | 4 | 5 | 6 | 7 |
| 2 | 0.86 | 34808.1 | 90.5 | 9.5 |  |  |  |  |  |
| 3 | 0.66 | 34551.4 | 23.5 | 69.9 | 6.6 |  |  |  |  |
| 4 | 0.67 | 34432.2 | 65.6 | 21.4 | 11.1 | 1.9 |  |  |  |
| 5 | 0.73 | 34249.1 | 14.3 | 55.3 | 25.4 | 3.2 | 1.8 |  |  |
| 6 | 0.74 | 34196.4 | 4.1 | 60.4 | 25.4 | 3.3 | 6.4 | 1.7 |  |
| 7 | 0.76 | 34179.0 | 24.9 | 0.7 | 3.3 | 4.5 | 59.6 | 5.5 | 1.5 |
