# Supplementary material for: Dynamic increase in myoglobin level is associated with poor prognosis in critically ill patients: a retrospective cohort study
Source: Front Med (Lausanne). 2024 Jan 8;10:1337403. doi: 10.3389/fmed.2023.1337403 (PMC10804859; doi:10.3389/fmed.2023.1337403)
Supplement: Supplementary file 2 [file Table_2.docx]

**Supplementary Table 2**

| **Num. of latent class** |  | **Proportion of probability (%)** | | |
| --- | --- | --- | --- | --- |
|  |  | **> 0.7** | **> 0.8** | **> 0.9** |
| 2 |  |  |  |  |
|  | Class 1 | 97.7 | 95.9 | 91.7 |
|  | Class 2 | 80.2 | 69.4 | 59.9 |
| 3 |  |  |  |  |
|  | Class 1 | 64.0 | 44.7 | 23.5 |
|  | Class 2 | 81.7 | 70.1 | 51.5 |
|  | Class 3 | 85.2 | 77.8 | 67.9 |
| 4 |  |  |  |  |
|  | Class 1 | 77.5 | 63.9 | 43.8 |
|  | Class 2 | 62.3 | 42.5 | 21.4 |
|  | Class 3 | 61.4 | 48.2 | 31.6 |
|  | Class 4 | 85.1 | 68.1 | 55.3 |
| 5 |  |  |  |  |
|  | Class 1 | 61.0 | 46.4 | 25.2 |
|  | Class 2 | 81.8 | 69.6 | 52.6 |
|  | Class 3 | 69.1 | 53.4 | 37.5 |
|  | Class 4 | 77.2 | 65.8 | 51.9 |
|  | Class 5 | 82.2 | 80.0 | 71.1 |
| 6 |  |  |  |  |
|  | Class 1 | 53.0 | 41.0 | 26.0 |
|  | Class 2 | 79.0 | 69.9 | 52.4 |
|  | Class 3 | 67.5 | 51.5 | 31.9 |
|  | Class 4 | 67.9 | 56.8 | 39.5 |
|  | Class 5 | 43.0 | 32.7 | 24.4 |
|  | Class 6 | 82.9 | 78.1 | 61.0 |
| 7 |  |  |  |  |
|  | Class 1 | 68.0 | 54.3 | 35.6 |
|  | Class 2 | 64.7 | 52.9 | 52.9 |
|  | Class 3 | 71.3 | 62.5 | 41.3 |
|  | Class 4 | 53.6 | 37.3 | 24.6 |
|  | Class 5 | 79.5 | 69.5 | 52.8 |
|  | Class 6 | 42.5 | 32.8 | 23.1 |
|  | Class 7 | 91.9 | 81.1 | 64.9 |

The percentages of subjects classified with a posterior probability above 0.7/0.8/0.9 means that the proportion of individuals assigned to each latent class with a posterior probability mean greater than 0.7/0.8/0.9. Larger proportion of probability means better classification.
