# Supplementary material for: Dynamic increase in myoglobin level is associated with poor prognosis in critically ill patients: a retrospective cohort study
Source: Front Med (Lausanne). 2024 Jan 8;10:1337403. doi: 10.3389/fmed.2023.1337403 (PMC10804859; doi:10.3389/fmed.2023.1337403)
Supplement: Supplementary file 3 [file Table_3.docx]

**Supplementary Table 3** Mean of posterior probability for each latent class

| Num. of latent class | | Mean of posterior probability for | | | |  |  |  |
| --- | --- | --- | --- | --- | --- | --- | --- | --- |
|  |  | Class 1 | Class 2 | Class 3 | Class 4 | Class 5 | Class 6 | Class 7 |
| 2 |  |  |  |  |  |  |  |  |
|  | Class 1 | 0.97 | 0.03 |  |  |  |  |  |
|  | Class 2 | 0.14 | 0.86 |  |  |  |  |  |
| 3 |  |  |  |  |  |  |  |  |
|  | Class 1 | 0.76 | 0.23 | 0.00 |  |  |  |  |
|  | Class 2 | 0.13 | 0.85 | 0.02 |  |  |  |  |
|  | Class 3 | 0.01 | 0.10 | 0.89 |  |  |  |  |
| 4 |  |  |  |  |  |  |  |  |
|  | Class 1 | 0.82 | 0.12 | 0.06 | 0.00 |  |  |  |
|  | Class 2 | 0.23 | 0.75 | 0.02 | 0.00 |  |  |  |
|  | Class 3 | 0.18 | 0.03 | 0.77 | 0.02 |  |  |  |
|  | Class 4 | 0.00 | 0.00 | 0.12 | 0.88 |  |  |  |
| 5 |  |  |  |  |  |  |  |  |
|  | Class 1 | 0.75 | 0.17 | 0.05 | 0.02 | 0.01 |  |  |
|  | Class 2 | 0.07 | 0.85 | 0.08 | 0.00 | 0.00 |  |  |
|  | Class 3 | 0.05 | 0.15 | 0.79 | 0.02 | 0.00 |  |  |
|  | Class 4 | 0.07 | 0.00 | 0.07 | 0.84 | 0.02 |  |  |
|  | Class 5 | 0.08 | 0.00 | 0.00 | 0.03 | 0.89 |  |  |
| 6 |  |  |  |  |  |  |  |  |
|  | Class 1 | 0.72 | 0.12 | 0.01 | 0.03 | 0.10 | 0.02 |  |
|  | Class 2 | 0.03 | 0.84 | 0.09 | 0.00 | 0.04 | 0.00 |  |
|  | Class 3 | 0.00 | 0.18 | 0.78 | 0.02 | 0.01 | 0.00 |  |
|  | Class 4 | 0.04 | 0.02 | 0.09 | 0.79 | 0.05 | 0.02 |  |
|  | Class 5 | 0.11 | 0.13 | 0.03 | 0.03 | 0.68 | 0.01 |  |
|  | Class 6 | 0.03 | 0.00 | 0.00 | 0.02 | 0.07 | 0.88 |  |
| 7 |  |  |  |  |  |  |  |  |
|  | Class 1 | 0.79 | 0.00 | 0.02 | 0.01 | 0.17 | 0.01 | 0.00 |
|  | Class 2 | 0.00 | 0.79 | 0.00 | 0.06 | 0.14 | 0.00 | 0.00 |
|  | Class 3 | 0.09 | 0.00 | 0.80 | 0.03 | 0.02 | 0.05 | 0.02 |
|  | Class 4 | 0.01 | 0.01 | 0.03 | 0.72 | 0.12 | 0.09 | 0.02 |
|  | Class 5 | 0.08 | 0.01 | 0.00 | 0.03 | 0.84 | 0.03 | 0.00 |
|  | Class 6 | 0.03 | 0.00 | 0.03 | 0.13 | 0.11 | 0.69 | 0.02 |
|  | Class 7 | 0.00 | 0.00 | 0.01 | 0.04 | 0.00 | 0.06 | 0.90 |

The LCTM algorithm will calculate the posterior probability of each person being assigned to each group. The classification table contains the mean of the posterior probability of belonging to each latent class over the subjects classified in each of the latent classes. As for the posterior probability matrix of setting different latent classes, the diagonal values lager than the others deem to be a better classification.
