# Supplementary material for: Dynamic increase in myoglobin level is associated with poor prognosis in critically ill patients: a retrospective cohort study
Source: Front Med (Lausanne). 2024 Jan 8;10:1337403. doi: 10.3389/fmed.2023.1337403 (PMC10804859; doi:10.3389/fmed.2023.1337403)
Supplement: Supplementary file 4 [file Table_4.docx]

**Supplementary Table 4** Biomarkers of myocardial injury between groups

| **Characteristic** | **Overall**, N = 2,448^1^ | **Steady**, N = 1,606^1^ | **Gradually decreasing**, N = 523^1^ | **Slowly rising**, N = 272^1^ | **Rapidly rising**, N = 47^1^ |  |
| --- | --- | --- | --- | --- | --- | --- |
| Standard cTnI of Day1 | -0.13 (-0.14, -0.11) | -0.13 (-0.14, -0.12) | -0.13 (-0.14, -0.11) | -0.12 (-0.13, -0.09) | -0.12 (-0.13, -0.11) |  |
| Standard cTnI of Day2 | -0.13 (-0.14, -0.11) | -0.13 (-0.14, -0.12) | -0.13 (-0.14, -0.11) | -0.12 (-0.13, -0.09) | -0.11 (-0.13, -0.06) |  |
| Standard cTnI of Day3 | -0.13 (-0.14, -0.12) | -0.13 (-0.14, -0.12) | -0.13 (-0.14, -0.12) | -0.12 (-0.13, -0.08) | -0.09 (-0.12, -0.03) |  |
| Standard CK-Mb of Day1 | -0.11 (-0.16, -0.01) | -0.14 (-0.16, -0.07) | -0.02 (-0.10, 0.14) | -0.06 (-0.14, 0.18) | 0.03 (-0.14, 0.30) |  |
| Standard CK-Mb of Day2 | -0.14 (-0.17, -0.07) | -0.15 (-0.17, -0.11) | -0.10 (-0.15, 0.03) | -0.07 (-0.13, 0.10) | 0.09 (-0.07, 0.56) |  |
| Standard CK-Mb of Day3 | -0.16 (-0.17, -0.11) | -0.16 (-0.17, -0.13) | -0.15 (-0.17, -0.09) | -0.08 (-0.14, 0.12) | 0.40 (0.07, 0.96) |  |
| Pro-BNP of Day1 | 700 (225, 2,706) | 806 (234, 3,002) | 417 (188, 1,426) | 1,001 (330, 3,497) | 1,435 (407, 3,144) |  |
| Pro-BNP of Day2 | 1,056 (405, 3,338) | 1,037 (372, 3,308) | 887 (414, 2,012) | 1,559 (624, 5,112) | 5,634 (1,328, 12,048) |  |
| Pro-BNP of Day3 | 1,234 (449, 3,406) | 1,234 (419, 3,371) | 1,016 (447, 2,406) | 1,849 (671, 5,810) | 4,736 (1,233, 11,554) |  |
| ^1^Median (IQR) | | | | | | |
| Since cTnI and CK-Mb have multiple test methods, the values of each test method were standardized, the standard values equal to (certain test value - corresponding mean)/corresponding standard deviation | | | | | | |
