# Supplementary material for: Dynamic increase in myoglobin level is associated with poor prognosis in critically ill patients: a retrospective cohort study
Source: Front Med (Lausanne). 2024 Jan 8;10:1337403. doi: 10.3389/fmed.2023.1337403 (PMC10804859; doi:10.3389/fmed.2023.1337403)
Supplement: Supplementary file 5 [file Table_5.docx]

**Supplementary table 5** The association between myoglobin levels and organ functions

| **Characteristic** | **Overall, N = 6,872^1^** | **Normal, N = 2,986^1^** | **Mild, N = 2,163^1^** | **Middle, N = 823^1^** | **Severe, N = 900^1^** | **p-value^2^** |
| --- | --- | --- | --- | --- | --- | --- |
| SOFA score^3^ | 8.0 (6.0, 9.0) | 7.0 (6.0, 9.0) | 8.0 (6.0, 9.0) | 9.0 (8.0, 10.0) | 10.0 (8.5, 11.0) | <0.001 |
| Respiratory SOFA | 2.0 (2.0, 2.0) | 2.0 (1.0, 2.0) | 2.0 (2.0, 2.0) | 2.0 (2.0, 2.0) | 2.0 (2.0, 3.0) | <0.001 |
| Circulatory SOFA | 1.0 (0.0, 2.0) | 1.0 (0.0, 2.0) | 1.0 (0.0, 2.0) | 1.0 (0.0, 2.0) | 2.0 (0.0, 2.0) | <0.001 |
| Renal SOFA | 0.0 (0.0, 0.0) | 0.0 (0.0, 0.0) | 0.0 (0.0, 0.0) | 0.0 (0.0, 1.0) | 0.0 (0.0, 2.0) | <0.001 |
| Hepatic SOFA | 0.0 (0.0, 1.0) | 0.0 (0.0, 1.0) | 0.0 (0.0, 1.0) | 1.0 (0.0, 2.0) | 1.0 (0.0, 2.0) | <0.001 |
| Cruor SOFA | 3.0 (2.0, 4.0) | 3.0 (3.0, 4.0) | 3.0 (3.0, 4.0) | 3.0 (2.0, 3.0) | 2.0 (2.0, 3.0) | <0.001 |
| ^1^n (%); Median (IQR) | | | | | | |
| ^2^Pearson's Chi-squared test; Kruskal-Wallis rank sum test | | | | | | |
| ^3^ SOFA score: without GCS score | | | | | | |
