# Supplementary material for: Dynamic increase in myoglobin level is associated with poor prognosis in critically ill patients: a retrospective cohort study
Source: Front Med (Lausanne). 2024 Jan 8;10:1337403. doi: 10.3389/fmed.2023.1337403 (PMC10804859; doi:10.3389/fmed.2023.1337403)
Supplement: Supplementary file 6 [file Table_6.docx]

**Supplementary table 6** The association between myoglobin levels and organ functions

| **Characteristic** | **Overall**, N = 2,448^1^ | **Steady**, N = 1,606^1^ | **Gradually decreasing,** N = 523^1^ | **Slowly rising**, N = 272^1^ | **Rapidly rising**, N = 47^1^ | **p-value**^2^ |
| --- | --- | --- | --- | --- | --- | --- |
| SOFA score^3^ | 8.0 (6.0, 9.0) | 7.0 (6.0, 9.0) | 8.0 (6.0, 9.0) | 9.0 (8.0, 10.0) | 10.0 (8.5, 11.0) | <0.001 |
| Respiratory SOFA | 2.0 (1.0, 3.0) | 2.0 (1.0, 3.0) | 2.0 (2.0, 3.0) | 2.0 (2.0, 3.0) | 3.0 (2.0, 3.0) | <0.001 |
| Circulatory SOFA | 2.0 (0.0, 2.0) | 2.0 (0.0, 2.0) | 2.0 (0.0, 2.0) | 2.0 (1.0, 2.0) | 2.0 (2.0, 2.0) | <0.001 |
| Renal SOFA | 0.0 (0.0, 1.0) | 0.0 (0.0, 1.0) | 0.0 (0.0, 1.0) | 1.0 (0.0, 2.0) | 2.0 (1.0, 2.0) | <0.001 |
| Hepatic SOFA | 1.0 (0.0, 2.0) | 0.0 (0.0, 2.0) | 1.0 (0.0, 2.0) | 2.0 (0.0, 2.0) | 2.0 (1.0, 4.0) | <0.001 |
| Cruor SOFA | 3.0 (2.0, 3.0) | 3.0 (2.0, 4.0) | 2.0 (2.0, 3.0) | 2.0 (2.0, 3.0) | 2.0 (1.0, 2.0) | <0.001 |
| ^1^n (%); Median (IQR) | | | | | | |
| ^2^Pearson's Chi-squared test; Kruskal-Wallis rank sum test | | | | | | |
| ^3^ SOFA score: without GCS score | | | | | | |
