# Supplementary material for: Dynamic increase in myoglobin level is associated with poor prognosis in critically ill patients: a retrospective cohort study
Source: Front Med (Lausanne). 2024 Jan 8;10:1337403. doi: 10.3389/fmed.2023.1337403 (PMC10804859; doi:10.3389/fmed.2023.1337403)
Supplement: Supplementary file 7 [file Data_Sheet_1.DOCX]

**Supplementary Statistical Methods**

**Latent class trajectory modelling**

Latent class trajectory modelling (LCTM) simplifies heterogeneous populations into more homogeneous clusters or classes. Random effects can potentially include to allow for individual variation within classes ^1^. LCTM uses maximum likelihood estimation and the probability of each individual being assigned to each class will be calculated separately. For each person, the class with the highest probability is the class to which he/she belongs. Meanwhile, LCTM is flexible enough to deal with different observation times between participants. In order to avoid overfitting, LCTM was only fitted using quadratic polynomials functions for myoglobin and missing values were not imputed in this study.

**Reasons for the choice of 4 groups**

Based on BIC and relative entropy, it seemed that the number of classes equaled to 5 was a better choice. However, the proportions of two classes were low and the model was not robust. If the number of classes was 3, although the proportion of each class seemed satisfactory, some important information provided by a few people whose myoglobin was extremely high would be lost. Thus, we selected the number of 4 groups finally.

**Restricted mean survival time**

RMST is defined as the area under Kaplan-Meier curve from 0 to $\tau$, where $\tau$ is the restricted time point. $\tau$ was set to 28-day in this study. RMST corresponds to the population parameter, while dependent variable for each individual is required in regression analysis. Pseudo value associates the population parameter RMST with individual covariates.

The *i*th person’s pseudo value is $\widehat{\theta_{i}}=n\int_{0}^{\tau} \widehat{S}(t)dt-(n-1)\int_{0}^{\tau} \widehat{S^{-i}}(t)dt$, where $n$ is the total sample size, $\int_{0}^{\tau} \widehat{S}(t)dt$ is the RMST based on the population, $\int_{0}^{\tau} \widehat{S^{-i}}(t)dt$ is the “leave-one-out” estimator of RMST ^2^. In the framework of pseudo value, the survival time of dead is penalized while the survival time of censored is prolonged, to some extent. The regression coefficients, $\beta$, can be estimated based on the generalized estimating equations and the corresponding variance could be obtained from the standard sandwich estimator. Owing that the RMST regression based on pseudo values does not need to satisfy the proportional hazard assumption and the regression coefficient can be explained as “the relative variation of life expectance due to a given factor within 28 days”, this approach works well for this study.

**References:**

1. Lennon H, Kelly S, Sperrin M, et al. Framework to construct and interpret latent class trajectory modelling. *BMJ Open.* 2018;8(7):e020683.

2. Andersen PK, Hansen MG, Klein JP. Regression analysis of restricted mean survival time based on pseudo-observations. *Lifetime Data Anal.* 2004;10(4):335-350.
