# Supplementary figures and images for: Dynamic increase in myoglobin level is associated with poor prognosis in critically ill patients: a retrospective cohort study
Source: Front Med (Lausanne). 2024 Jan 8;10:1337403. doi: 10.3389/fmed.2023.1337403 (PMC10804859; doi:10.3389/fmed.2023.1337403)

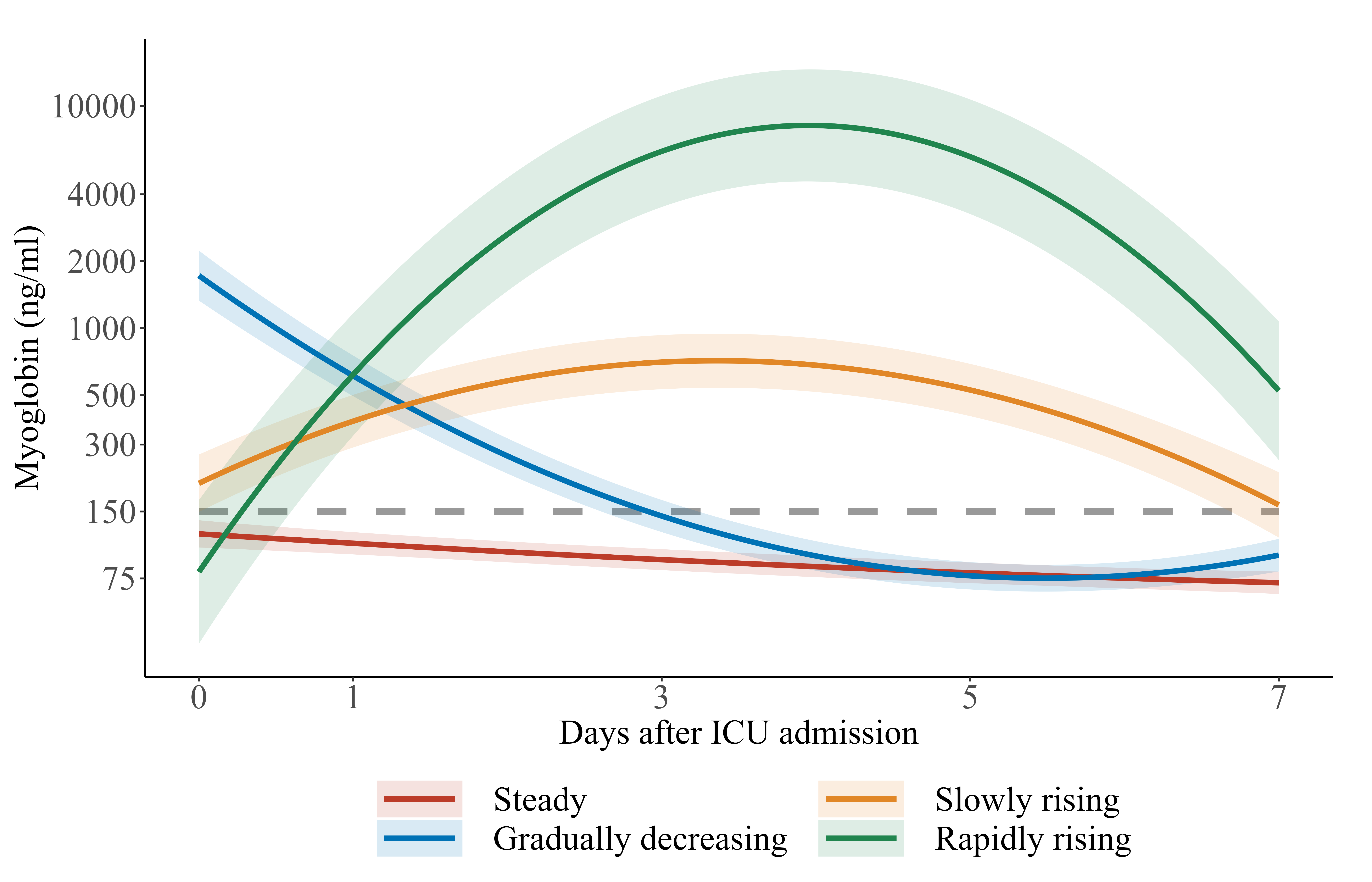

Supplement: SUPPLEMENTARY FIGURE 1 — Trajectories of myoglobin for sensitivity analysis. Individuals whose survival time was over than 72 hours were preserved in the sensitivity analysis. The gray dotted line represents 150 ng/ml. [file Image_1.TIFF]

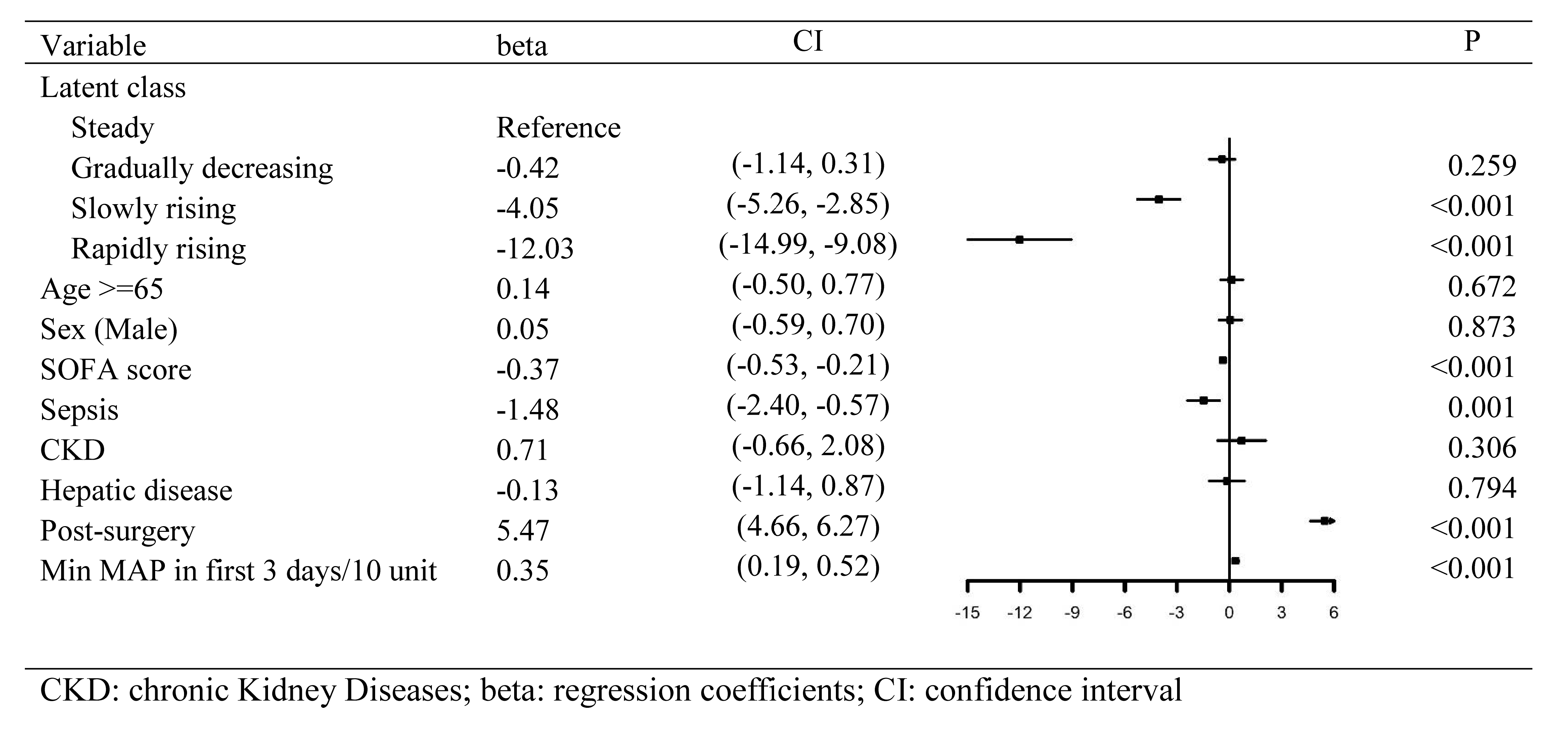

Supplement: SUPPLEMENTARY FIGURE 4 — Forest plot of multivariate regression results calculated by restricted mean survival time based on pseudo values after missing value imputation. [file Image_4.TIF]
